# A randomised controlled trial of a blended learning education intervention for teaching evidence-based medicine

**DOI:** 10.1186/s12909-015-0321-6

**Published:** 2015-03-10

**Authors:** Dragan Ilic, Rusli Bin Nordin, Paul Glasziou, Julie K Tilson, Elmer Villanueva

**Affiliations:** 1 Department of Epidemiology & Preventive Medicine, School of Public Health & Preventive Medicine, Level 6, The Alfred Centre, 99 Commercial Rd, Melbourne, VIC 3004 Australia; 2 Jeffrey Cheah School of Medicine and Health Sciences, Monash University, Johor Bahru, Malaysia; 3 Faculty of Health Sciences and Medicine, Bond University, Robina, Australia; 4 Division of Biokinesiology and Physical Therapy, University of Southern California, Los Angeles, USA; 5 Gippsland Medical School, Monash University, Churchill, Australia

**Keywords:** Evidence based medicine, Assessment, Medical education, Blended learning

## Abstract

**Background:**

Few studies have been performed to inform how best to teach evidence-based medicine (EBM) to medical trainees. Current evidence can only conclude that any form of teaching increases EBM competency, but cannot distinguish which form of teaching is most effective at increasing student competency in EBM. This study compared the effectiveness of a blended learning (BL) versus didactic learning (DL) approach of teaching EBM to medical students with respect to competency, self-efficacy, attitudes and behaviour toward EBM.

**Methods:**

A mixed methods study consisting of a randomised controlled trial (RCT) and qualitative case study was performed with medical students undertaking their first clinical year of training in EBM. Students were randomly assigned to receive EBM teaching via either a BL approach or the incumbent DL approach. Competency in EBM was assessed using the Berlin questionnaire and the ‘Assessing Competency in EBM’ (ACE) tool. Students’ self-efficacy, attitudes and behaviour was also assessed. A series of focus groups was also performed to contextualise the quantitative results.

**Results:**

A total of 147 students completed the RCT, and a further 29 students participated in six focus group discussions. Students who received the BL approach to teaching EBM had significantly higher scores in 5 out of 6 behaviour domains, 3 out of 4 attitude domains and 10 out of 14 self-efficacy domains. Competency in EBM did not differ significantly between students receiving the BL approach versus those receiving the DL approach [Mean Difference (MD)=−0.68, (95% CI–1.71, 0.34), p=0.19]. No significant difference was observed between sites (p=0.89) or by student type (p=0.58). Focus group discussions suggested a strong student preference for teaching using a BL approach, which integrates lectures, online learning and small group activities.

**Conclusions:**

BL is no more effective than DL at increasing medical students’ knowledge and skills in EBM, but was significantly more effective at increasing student attitudes toward EBM and self-reported use of EBM in clinical practice. Given the various learning styles preferred by students, a multifaceted approach (incorporating BL) may be best suited when teaching EBM to medical students. Further research on the cost-effectiveness of EBM teaching modalities is required.

**Electronic supplementary material:**

The online version of this article (doi:10.1186/s12909-015-0321-6) contains supplementary material, which is available to authorized users.

## Background

Evidence-based medicine (EBM), also referred to as evidence-based practice (EBP), has been adopted as a core unit across many medical schools worldwide [1,2], with a particular focus in Australian Universities [3,4]. EBM is based on the principle that informed medical decision making is achieved by integrating the best available evidence with clinical expertise and patient values [5]. For clinicians to be competent in EBM, they must be able to understand and apply the following EBM steps (ask, acquire, appraise, apply and assess) in clinical practice:

1. Ask a clinical question that is constructed using the PICO (patient, intervention, comparison, outcome) framework;
2. Acquire evidence via a systematic and efficient search of the literature;
3. Appraise the evidence through application of critical appraisal techniques;
4. Apply the evidence to the clinical scenario; and
5. Assess the EBM process as it relates to the clinical context [5].

Each step within the EBM process requires a different level of competency (i.e. integration of knowledge, skill, attitude and behaviour) from the user [6]. Achieving a high level of competency in EBM can only be achieved when the user is able to effectively undertake all five steps, which incorporate adequate levels of knowledge, skills, attitude and behavioural elements [6]. Achieving competency in EBM not only provides users with the ability to make informed medical decisions, but also the skills required to be life-long learners in their discipline.

Due to the multifaceted nature of knowledge and skills required to be competent in EBM, it is evident that teaching EBM should integrate core knowledge with clinical practical activities in a bid to improve competency in EBM [7]. EBM may be taught across a variety of modes including lectures, tutorials, mini-courses, online, problem based or self-directed learning [7]. However, limited evidence currently exists in order to inform educators as to the most effective method of teaching and increasing user competency in EBM.

A 2004 systematic review identified two randomised controlled trials (RCTs) and seven non-RCTs that examined the effectiveness of different teaching modalities in EBM across post-graduate students [8]. The authors of that review concluded that standalone teaching improved student knowledge, but not skills, attitudes or behaviour in EBM. Conversely, evidence from the non-RCTs indicated that integrating teaching of EBM with clinical activities (i.e. blended learning) was associated with improvements across all four domains (i.e. knowledge, skills, attitudes and behaviour) [8]. A more recent systematic review examined the impact of different teaching modalities on medical trainees [9]. Based on 9 RCTs identified, the authors concluded that any form of teaching, including lecture, tutorial, self-directed, online, problem-based, uni and multidisciplinary, was associated with an increase in EBM competency. However, no single intervention was identified as being significantly better than others at increasing EBM competency.

Student learning styles, infrastructure or other organisational issues may all dictate how an EBM course is implemented. EBM requires mastery across a variety of disparate disciplines including epidemiology, biostatistics, informatics and information literacy. Given the different learning outcomes across these disciplines, a multifaceted approach is required to teach a multidimensional discipline such as EBM [10]. Blended learning (BL) attempts to create an optimal learning environment by blending a variety of learning approaches (lecture, tutorial, online, problem-based and clinical) to account for different learning styles and requirements [10]. Learning styles in this instance refers to the concept that individuals differ with respect to what mode of instruction or study is most effective for them in processing, absorbing and retaining information [11].

Few RCTs have evaluated the effectiveness of the BL model in medicine. The majority of studies performed to date have focussed on clinical disciplines within medicine and have reported an increase in student self-efficacy, knowledge and self-directed learning [12-14]. The first controlled trial examining BL within the EBM context was performed in a small cohort of graduate-entry medical students and identified no difference in EBM competency between students receiving the BL approach and those receiving a didactic learning (DL) approach [3]. However, students receiving the BL model reported significantly greater self-efficacy and confidence in their EBM competency and ability to translate theory into practice.

EBM aims to promote informed medical decision making, yet currently, there is a lack of evidence to inform educators and learners as to the most effective methods of teaching EBM to medical students. The aim of this study was to conduct the first RCT to examine the effectiveness of implementing a BL versus DL approach of teaching EBM to medical students. This study also aimed to examine students’ self-efficacy, perceptions and attitudes toward EBM teaching delivered through a BL approach.

## Methods

### Design

We used a mixed methods design, incorporating a RCT and a focus group follow up assessment. Use of this mixed methods approach provides an opportunity for quantitative data to inform the effectiveness of the intervention, whilst the qualitative data contextualise quantitative results by addressing issues of ‘how’ and ‘why’ [15]. The methods of this study have been previously published as a protocol [16].

### Settings and participants

A multi-campus study was performed with medical students enrolled in the MBBS course at Monash University. Monash University runs undergraduate and graduate-entry MBBS programs at both its campuses in Australia and Malaysia. During the first clinical teaching year of the course, students are assigned to one of seven metropolitan hospitals, or six rural, hospitals in Australia (with one site in Malaysia). Participants were third year medical students, who were all entering their first year of clinically-based training and first year of formal EBM training.

### Recruitment for RCT

As part of their EBM training, students are randomly allocated to a tutorial group consisting of approximately 20–25 students in a group. Tutorial groups were randomised to receive EBM teaching adopting a BL or a DL approach. Students not wishing to participate in the study were taught via the DL approach (the current approach to teaching EBM) and did not complete any outcome assessments.

### Randomisation

Students were randomised according to their tutorial group (i.e. cluster) by a researcher independent to the study utilising a simple cluster randomisation procedure (computerised random numbers). All students were provided with access to the BL materials at the conclusion of the study period to ensure parity between groups for upcoming examinations.

### Control

Students randomised to the control group received the DL model. The DL model consisted of a 10 two-hour teaching sessions in which formal EBM concepts are delivered by a tutor/lecturer to students. Teaching sessions commence with a formal presentation, which is followed by students performing a small group activity to consolidate their learning. This small group activity is commonly a critical appraisal of an article relating to the study design discussed by the lecturer/tutor incorporating elements of therapy, harm, prognosis and diagnosis.

### Intervention

Students randomised to the intervention group received the same theoretical concepts taught in the control group, but in a BL approach. The BL approach to teaching EBM integrated (i) classroom activities (lecture/tutorial) with (ii) online and (iii) mobile learning. The online component was provided through specific resources delivered via the Monash library website [17], as well as specifically designed online lectures, made available through YouTube, which students were asked to view prior to attending the respective two hour teaching block [17,18]. The mobile learning component was delivered on the wards, when students were interacting with patients during their existing day-to-day ‘bedside teaching’ schedule. During the mobile learning, students were required to take a detailed medical history from the patient, as they normally would during their ‘bedside’ teaching. Students would then apply the relevant week’s EBM content, before presenting their patient case during the next EBM tutorial. The methodology for this intervention was previously piloted [3]. Further details about the DL and BL approaches can be found in the protocol of this study [16].

### Outcome measures

Student competency in EBM was assessed by a blinded outcome assessor, one-month post-teaching activities, using the validated Berlin Questionnaire and ACE tool [19,20]. The Berlin Questionnaire is a 15-point multiple-choice item questionnaire that has been developed and validated to measure medical professionals’ knowledge and skills in EBM [19]. The ACE tool is a 15-point dichotomous-choice item questionnaire that has been developed and validated to measure medical students’ knowledge and skills in EBM [20]. Student self-efficacy was assessed using the Evidence-Based Practice Question (EBPQ) [21]. The EBPQ is a self-reported measure of implementation of EBM, with measures relating to self-efficacy, behaviour and attitudes toward EBM.

### Blinding

Due to the educational nature of the intervention, it was not possible to blind either the educators or the students. The outcome assessor and data analyst were kept blinded to allocation.

### Analyses

#### Sample size calculation

To detect a 50% difference in EBM competency (α=0.05, β=0.80, σ=2.8) between groups, it was determined that a minimum of 120 students per arm (40 from each of metropolitan Melbourne (undergraduate), rural Victoria (graduate) and Monash Malaysia (international)), were required for recruitment.

#### Statistical analyses

Quantitative data was analysed using the principle of intention-to-treat. Mean differences (MD) in EBM competency and self-efficacy between intervention and control groups was explored using Student’s *t*-test for parametric and Mann–Whitney *U*-test for non-parametric data. Differences between intervention/control groups and student type were explored using one and two-way ANOVAs.

### Recruitment for focus groups

At the conclusion of the RCT, students from the 13 Australian hospital sites, and one Malaysian site, who received the intervention were invited to participate in focus groups using a convenience sampling approach [22]. Students were required to provide written consent prior to their participation in the group.

### Data collection

All focus groups were homogeneous in their composition (i.e. Australian metropolitan, rural or Malaysian sites). All focus groups at the Australian sites were performed by a facilitator independent to the study, whilst another facilitator was required to lead focus groups in Malaysia. All focus group discussions were guided by a semi-structured interview schedule (Additional file 1). Each focus group was comprised of up to six students, and were digitally recorded and transcribed verbatim at the conclusion of the focus groups. Focus groups were run until the point of theoretical saturation, whereby no further novel ideas were generated through discussion.

### Data analysis

Transcripts from all focus groups were analysed independently by two researchers (DI and another independent academic) using thematic analysis [23]. Thematic analysis consisted of a six step approach including; (i) familiarization of the data, (ii) generation of preliminary codes, (iii) searching for themes from the preliminary codes, (iv) creation of a thematic map, (v) specific defining of themes, and (vi) final analysis [24].

### Ethics

Ethical approval for this study was obtained from the Monash University Human Research Ethics Committee. Ethics approval was applicable to all Australian and Malaysian sites participating in the study.

## Results

**Figure 1:**
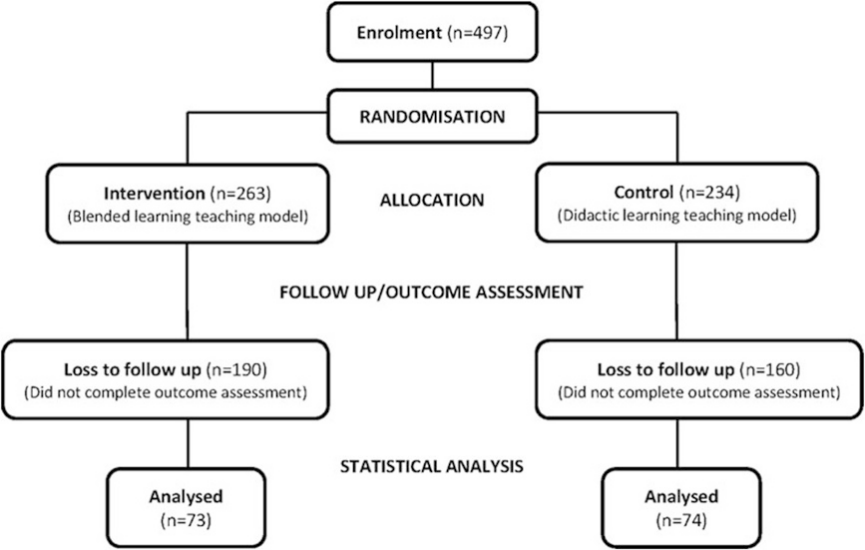
**Flow diagram of randomised controlled trial.**

A total of 497 students were eligible and enrolled for participation in one of the two learning approaches. A total of 147 (30%) (45 graduate-entry and 102 undergraduate-entry) students completed the Berlin Questionnaire and ACE tool (Figure 1). The remaining 350 students declined to complete the outcome assessment. Of the 147 students completing the outcome assessment, 63 students were placed at an Australia metropolitan hospital, 45 at an Australian rural hospital and 39 at a Malaysian-based hospital. From the 147 students completing the outcome measures, 82 (56%) students also completed the EBPQ outcome measure. A further 29 students participated in 6 focus group discussions (10 students from the Malaysian campus participated in 2 focus group discussions, 7 students from the graduate-entry program participated in 2 focus group discussions and 12 students from the Australian metropolitan-based hospitals participated in 2 focus group discussions).

**Figure 2:**
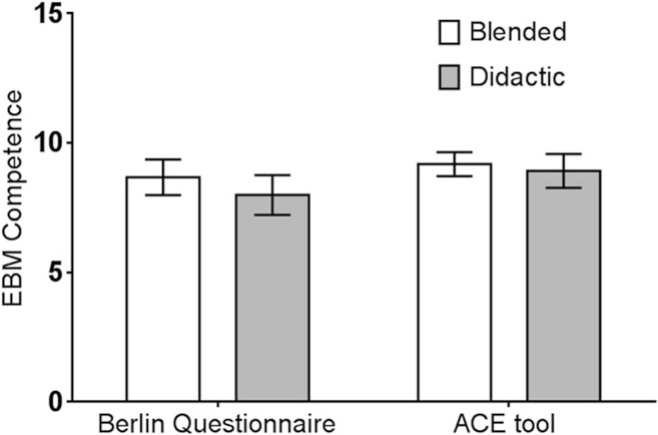
**Comparison of EBM competency across students randomised to blended learning or didactic learning methodologies.** EBM competency is assessed using the Berlin Questionnaire and the ACE tool (mean score ± 95% confidence interval).

**Figure 3:**
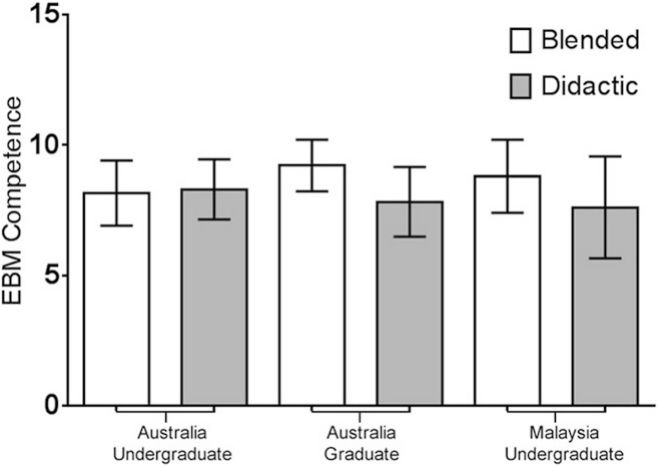
**Comparison of EBM competency across students randomised to blended learning or didactic learning methodologies.** EBM competency is assessed using the Berlin Questionnaire (mean score ± 95% confidence interval).

**Figure 4:**
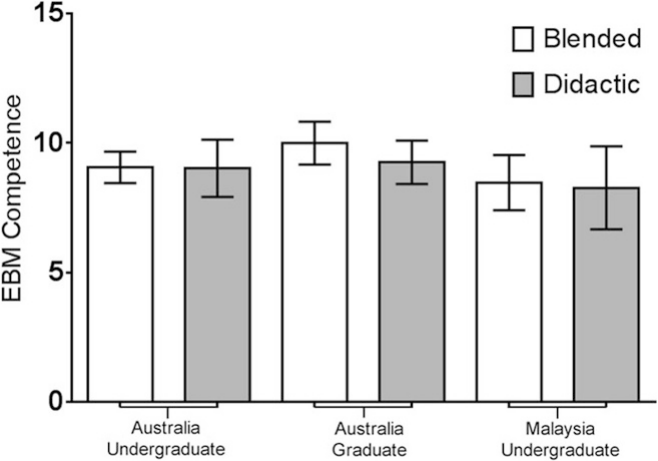
**Comparison of EBM competency across students randomised to blended learning or didactic learning methodologies.** EBM competency is assessed using the ACE tool (mean score ± 95% confidence interval).

No significant difference in EBM competency was observed between students undertaking the EBM course using the BL approach compared to students undertaking the DL approach. This outcome was consistent regardless of whether outcomes were assessed via the Berlin Questionnaire [MD=−0.68, (95% CI–1.71, 0.34), p=0.19] or the ACE tool [MD=−2.5, (95% CI–1.05, 0.53), p=0.52] (Figure 2). No significant difference in EBM competency was observed between Australian-based undergraduate, Malaysian-based undergraduate, or Australian-based graduate-entry students (Berlin Questionnaire p=0.89; ACE tool p=0.09) (Table 1). Further analysis demonstrated no significant difference in EBM competency between students within those sites regarding method of EBM delivery (Berlin Questionnaire p=0.58; ACE tool p=0.26) (Figures 3 and 4).

**Table 1:** **EBM competency across Australian undergraduate, graduate and Malaysian undergraduate cohorts**

Cohort comparison	Mean difference (95% CI)
*EBM competency as measured by the Berlin Questionnaire*	
Australian undergraduate vs Australian graduate	−0.27 (−1.75 to 1.20)
Australian undergraduate vs Malaysian undergraduate	−0.02 (−1.55 to 1.51)
Australian graduate vs Malaysian undergraduate	0.25 (−1.39 to 1.90)
**Cohort comparison**	**Mean rank difference**
*EBM competency as measured by the ACE tool*	
Australian undergraduate vs Australian graduate	−6.467
Australian undergraduate vs Malaysian undergraduate	13.12
Australian graduate vs Malaysian undergraduate	19.58

**Figure 5:**
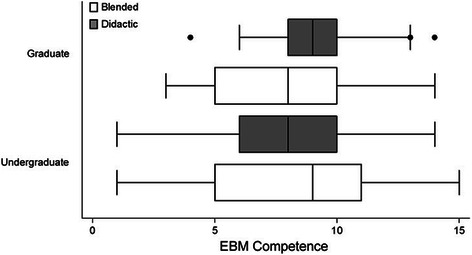
**Comparison of EBM competency across graduate and undergraduate students randomised to blended learning or didactic learning methodologies.** EBM competency is assessed using the Berlin Questionnaire (mean score ± 95% confidence interval).

**Figure 6:**
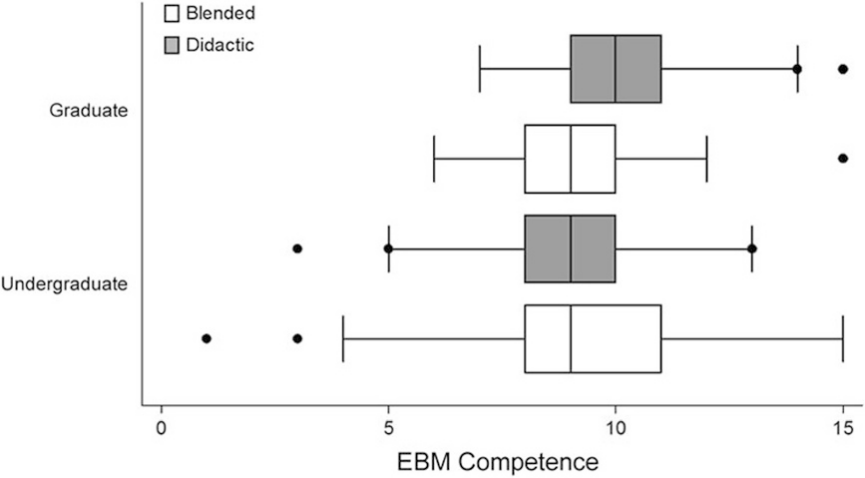
**Comparison of EBM competency across graduate and undergraduate students randomised to blended learning or didactic learning methodologies.** EBM competency is assessed using the ACE tool (mean score ± 95% confidence interval).

No significant difference in EBM competency was observed between undergraduate versus graduate students. This outcome was consistent regardless of whether outcomes were assessed via the Berlin Questionnaire [MD=−0.26, (95% CI–1.38, 0.85), p=0.64] or the ACE tool (median difference=0, p=0.12). Further analysis demonstrated no significant difference in EBM competency between undergraduate and graduate student cohorts regarding method of EBM delivery, be it via the BL or DL approach (Berlin Questionnaire p=0.36; ACE tool p=0.44) (Figures 5 and 6).

Perceived self-efficacy, attitudes and behaviour toward EBM was significantly higher in students who received the BL approach (Table 2). Students who received the BL approach had significantly higher scores in 5 out of 6 behaviour domains, 3 out of 4 attitude domains and 10 out of 14 self-efficacy domains. Students who received the BL approach also reported significantly higher scores relating to the implementation of the 5 steps relating to EBM (as identified through questions 1–5 on the EBPQ).

**Table 2:** **Self-efficacy, attitudes and behaviour across students randomised to blended and didactic learning approaches**

Question	Blended learning (Mean ± SD) n=44	Didactic learning (Mean ± SD) n=38	Mean difference (95%CI)
***Practice of evidence-based practice***			
1. How often have you formulated a clearly answerable question as the beginning of the process towards filling an information gap?	6.22 ± 0.16	4.21 ± 0.33	2.01 (1.29 to 2.73)*
2. How often have you tracked down the relevant evidence once you have formulated the question?	6.13 ± 0.20	5.05 ± 0.24	1.08 (0.45 to 1.7)*
3. How often have you critically appraised any literature you have discovered?	5.63 ± 0.27	4.26 ± 0.32	1.37 (0.52 to 2.2)*
4. How often have you integrated the evidence you have found with your activities?	5.72 ± 0.27	4.10 ± 0.28	1.62 (0.82 to 2.41)*
5. How often have you evaluated the outcomes of your EBCP practice?	5.50 ± 0.28	3.26 ± 0.36	2.24 (1.31 to 3.15)*
6. How often have you shared information that you’ve gathered with colleagues?	5.50 ± 0.34	4.78 ± 0.32	0.72 (−0.23 to 1.65)
***Attitude towards evidence-based practice***			
7. New evidence is so important that I make the time in my work schedule	5.09 ± 0.32	3.73 ± 0.21	1.36 (0.56 to 2.14)*
8. I welcome questions on my practice	6.09 ± 0.22	5.31 ± 0.18	0.78 (0.19 to 1.35)*
9. Evidence based practice is fundamental to professional practice	6.63 ± 0.12	6.15 ± 0.14	0.48 (0.09 to 0.85)*
10. My practice has changed because of evidence I have found	5.68 ± 0.21	5.10 ± 0.23	0.58 (−0.04 to 1.20)
***Knowledge/skills associated with evidence-based practice***			
11. How would you rate your research skills?	5.09 ± 0.21	3.73 ± 0.19	1.36 (0.78 to 1.92)*
12. How would you rate your IT skills?	5.68 ± 0.24	4.47 ± 0.22	1.21 (0.54 to 1.87)*
13. How would you rate your ability to monitor and review your EBCP skills?	5.22 ± 0.26	3.73 ± 0.20	1.49 (0.80 to 2.17)*
14. How would you rate your ability to convert your information needs into a clinical question?	5.95 ± 0.19	4.68 ± 0.15	1.27 (0.76 to 1.77)*
15. How would you rate your awareness of major information types and sources?	5.27 ± 0.25	5.26 ± 0.13	0.01 (−0.59 to 0.61)
16. How would you rate your ability to identify gaps in your professional practice?	4.81 ± 0.19	4.36 ± 0.20	0.45 (−0.12 to 1.02)
17. How would you rate your knowledge of how to retrieve evidence?	5.31 ± 0.24	4.94 ± 0.18	0.37 (−0.25 to 1.00)
18. How would you rate your ability to analyse critically evidence?	5.09 ± 0.20	4.15 ± 0.16	0.94 (0.40 to 1.46)*
19. How would you rate your ability to determine how valid (close to the truth) the material is?	5.00 ± 0.26	4.05 ± 0.19	0.95 (0.27 to 1.61)*
20. How would you rate your ability to determine how useful (clinically applicable) the material is?	5.68 ± 0.18	4.57 ± 0.12	1.11 (0.64 to 1.56)*
21. How would you rate your ability to apply information to individual cases?	5.40 ± 0.18	4.78 ± 0.17	0.62 (0.10 to 1.13)*
22. How would you rate your sharing of ideas and information with colleagues?	5.18 ± 0.27	4.84 ± 0.19	0.34 (−0.35 to 1.03)
23. How would you rate your dissemination of new ideas about care to colleagues?	5.31 ± 0.27	3.89 ± 0.16	1.42 (0.75 to 2.09)*
24. How would you rate your ability to review your own practice?	5.22 ± 0.24	4.15 ± 0.14	1.07 (0.47 to 1.66)*

*p-value < 0.05.

Analysis of focus group discussions with students identified four themes relating to (i) preferred learning approach, (ii) perceptions of the blended learning approach, (iii) perceptions of the didactic learning approach, and (iv) barriers and enablers to teaching EBM.

### Preferred learning approach

Students preferred an integrated learning approach that facilitated different learning styles. DL, whether it is delivered as a lecture or online multimedia resource, was preferred for the acquisition of ‘facts’ or foundation information (e.g. gross anatomy in clinical medicine and research methodologies in EBM). Students were amenable to engaging in self-directed DL approaches (e.g. viewing YouTube clips rather than attending a lecture). Workshops and small group activities were preferred for the acquisition of skills, be it clinical or EBM. The interactive nature of small group work facilitated greater student engagement with the content and provided motivation to apply learnt skills in the clinical context.

“I like the interactive style with workshops and small groups… you get your hands dirty, with lectures you tend to zone out…”

### BL approach

Students were positive about using the BL approach in teaching and learning about EBM. The three-step approach of (i) self-directed learning through viewing online multimedia presentations, (ii) discussion and activities in class, and (iii) application in practice was positively received by students. Students receiving the BL approach found the content useful, engaging and well-targeted to their level of competency. Students suggested that the BL approach could be strengthened by introducing a journal club approach to small group activities early in the curriculum, providing an opportunity to learn in a group environment before greater emphasis was placed on individual self-directed learning in the latter aspect of the curriculum.

*“It was like someone thinking out aloud, someone who knew what they were doing, so understood the thought process (…behind teaching EBM to students)”.*

### DL approach

Students receiving the DL approach perceived the EBM content delivered as dense and dry. Students suggested that this approach only promoted superficial learning of the EBM content, sufficient only for adequate completion of assessment tasks. Students described the variation in perceived ability of different tutors across sites to engage with students and demonstrate the applicability of the material and EBM to clinical practice. Upon completion of the trial, students in the DL group were able to view the YouTube clips–all suggested that the provision of such online didactic presentations would provide greater engagement with students and perceived parity of teaching across sites.

*“…the clarity of the information presented in the videos compared with that presented by the tutors was miles apart”.*

### Barriers to teaching EBM

The most common barrier reported in implementing the BL approach was the method of implementation by tutors across the 14 teaching hospitals involved in this study. Although students were required to view the online clips prior to the tutorials, some sites would show the clips in-class, to appease students who did not do the pre-tutorial activities. This would negatively impact upon the time allocated for in-class activities. Students involved in this study were experiencing the first year of teaching in a clinical environment. Many students reported a disconnect between the teaching of EBM and perceived application in their current clinical teaching. Students suggested that teaching EBM may have greater clinical value with students after a longer exposure to clinical teaching.

“We are still learning to walk and yet they want us to run (in terms of applying EBM to the clinical context)”.

## Discussion

This study was the first RCT to examine the value of teaching EBM to medical students via a BL approach. Our findings demonstrate equivalence in student EBM competency regardless of whether teaching was implemented using a BL or DL approach. This equivalence in student EBM competency was not significantly different between undergraduate and graduate-entry students, or amongst Australian and Malaysian based students. Perceived self-efficacy, attitudes and behaviour were significantly higher in students receiving the BL approach.

Findings from this RCT support pilot findings from a non-randomised study of graduate-entry medical students, which demonstrated no significant difference in EBM competency between students regardless of teaching method (BL or DL) utilised [3]. Findings from our trial support systematic review evidence that suggests equivalence amongst teaching modalities when teaching EBM, be it DL, BL, uni or multi-disciplinary [9]. It further supports evidence from non-randomised studies in post-graduate medical students that suggest that integrating EBM teaching with clinical activities is associated with relative increases in knowledge, skills, attitudes and behaviours.

The quantitative results would suggest no difference in learner competency in EBM, which may in part be attributed to the nature of the assessments, of which the majority of items assess cognitive knowledge, rather than direct application in a clinical context. The qualitative findings would suggest that a BL approach to teaching EBM is more successful at improving student behaviours and attitudes toward EBM. A student’s perceived relevance of EBM, application (both seen and actual) and clinical maturity may influence competency in EBM [25-28]. Students who received the BL approach in this study had significantly higher (albeit self-reported) levels of self-efficacy, behaviour and attitude toward EBM compared to those that received the DL approach. Our qualitative findings demonstrated a distinct student preference for a BL approach (for both clinical and EBM related teaching), since this mode of teaching was perceived to provide better student engagement with key theoretical and practical components [3].

A variety of barriers may prevent the implementation of evidence into practice, including perceived relevance, awareness or opportunity [29]. Our study has demonstrated that students receiving the BL approach were significantly more likely to be implementing the five key steps of EBM at this early stage of their clinical careers. Implementing an EBM program during the first year in which students are exposed to the clinical environment may facilitate a greater link between the theory of EBM and its use in clinical practice. Students in this study highlighted the potential disconnect between what they were learning and practicing in the clinical environment. Such disconnect may be attributed to a lack of clinical maturity, or lack of mentorship from senior clinical staff who may themselves not be practitioners of EBM [25,30].

Use of BL in clinical medicine is becoming more prominent, as educators use lectures to disseminate information required for foundation learning, whilst small group activities (including problem-based learning), online learning (including self-directed) and patient-centred learning consolidate theory with practice [12-14]. Given the multi-disciplinary nature of EBM, use of a BL approach is appropriate. As demonstrated in our study, lectures and online presentations should be used to present foundation knowledge (e.g. research methods, information literacy and critical appraisal techniques), whilst small group activities should be used to consolidate skills including critical appraisal and application to the clinical scenario. Journal clubs are commonly used as an interactive method of teaching and practicing EBM in clinical practice [31]. The effectiveness of journal clubs in increasing EBM competency remains unclear, and further research is required to ascertain how they could best be integrated in EBM teaching to further promote learner competency [31].

This study was the first to utilise a RCT methodology to examine the effectiveness of BL in EBM. The use of qualitative data provided critical contextualisation of quantitative results and further rigour to the study through triangulation of data. The study was successfully implemented across 14 teaching hospitals in Australia and Malaysia. Competency in EBM was assessed by two validated and psychometrically tested tools, although the self-report questionnaire has not been previously validated. Whilst students were compliant in their uptake of the teaching intervention, less than 30% completed the outcome assessments. This unexpected low completion rate may have underpowered the RCT. Students with a higher ability and affiliation with the teaching content are more likely to respond to survey requests and assessments in projects [32]. The low completion rate of outcome assessments for this RCT increases the risk of bias in the study regarding the generalisability of results due to the greater likelihood of students with a higher affiliation to EBM completing the outcome assessments. No estimate of how long students, on average, engaged with the online resources.

Students in the focus group discussions highlighted the variability in how some tutors incorporated the teaching materials within the BL approach. For example, some tutors repeated YouTube presentations during class time, thereby impacting upon the need for student self-directed learning outside of class. Future implementation of a BL approach for EBM will require strategies for training educators in how they effectively teach using a BL approach. Few studies have examined the cost-effectiveness of education strategies in medicine [33,34]. The current evidence base would suggest equivalence in teaching strategies for EBM, yet no published evidence on the cost associated with these interventions is currently available. Better understanding of start-up and on-going costs will provide educators with valuable information that may influence the type of educational strategy used to teach EBM. Information about cost-effectiveness and value is important given that start-up costs with the production of e-learning resources are initially high, but dissipate with continual use.

## Conclusion

The current EBM demonstrates equivalence between adopting a BL versus a DL approach to teaching EBM to medical students. However, medical students receiving a BL approach reported greater perceived self-efficacy and application of EBM in the clinical environment. Future research should focus on costs associated with teaching EBM in order to identify a cost-effective strategy for teaching EBM.

## Additional file

Additional file 1:
**Interview schedule used to guide focus group discussions.**
