# Supplementary material for: A randomised controlled trial of a blended learning education intervention for teaching evidence-based medicine
Source: BMC Med Educ. 2015 Mar 10;15:39. doi: 10.1186/s12909-015-0321-6 (PMC4358913; doi:10.1186/s12909-015-0321-6)
Supplement: Additional file 1: — Interview schedule used to guide focus group discussions. [file 12909_2015_321_MOESM1_ESM.docx]

**Additional file 1**

Interview schedule used to guide focus group discussions.

1. What type of material do you prefer to be taught in (and why)?
   1. Lecture format
   2. Tutorial format
   3. Small groups
   4. Self-directed
   5. Online
   6. Workshops
2. How is EBM best delivered (probe reasoning for responses)?
   1. Lecture
   2. Tutorial
   3. Online
   4. Combination etc…
3. Would ‘flip’ learning suit the teaching of EBM (i.e. pre-loading information before class)?
4. Should EBM teaching be blended with
   1. Bed-side teaching
   2. PBL (problem based learning)
   3. Other…
5. What aspects of the EBM program work best? (Probe responses)
6. How can the teaching of EBM be improved? (Probe responses)
